# Purification and Characterization of Antibodies in Single-Chain Format against the E6 Oncoprotein of Human Papillomavirus Type 16

**DOI:** 10.1155/2018/6583852

**Published:** 2018-05-20

**Authors:** F. Verachi, Z. Percario, P. Di Bonito, E. Affabris, C. Amici, L. Accardi

**Affiliations:** ^1^Department of Biology, University of Rome Tor Vergata, Rome, Italy; ^2^Department of Infectious Diseases, Italian National Institute of Health, Rome, Italy; ^3^Department of Science, Roma Tre University, Rome, Italy

## Abstract

In Human Papillomaviruses- (HPV-) associated carcinogenesis, continuous expression of the E6 oncoprotein supports its value as a potential target for the development of diagnostics and therapeutics for HPV cancer. We previously reported that the I7 single-chain antibody fragment (scFv) specific for HPV16 E6, expressed as an intrabody by retroviral system, could inhibit significantly the growth of cervical cancer cells* in vitro *and was even able to reduce tumor development in experimental HPV-related cancer models. Nevertheless, for the development of therapeutic tools to be employed in humans, it is important to achieve maximum safety guarantee, which can be provided by the protein format. In the current study, two anti-16E6 scFvs derived from I7 were expressed in* E. coli *and purified in soluble form by affinity chromatography. Specificity, sensitivity and stability in physiologic environment of the purified scFvs were demonstrated by binding studies using recombinant 16E6 as an antigen. The scFvs functionality was confirmed by immunofluorescence in cervical cancer cells, where the scFvs were able to recognize the nuclear E6. Furthermore, an antiproliferative activity of the scFvI7nuc delivered in protein format to HPV16-positive cell lines was observed. Our results demonstrate that functional anti-16E6 scFvs can be produced in* E. coli*, suggesting that such purified antibodies could be used in the diagnosis and treatment of HPV-induced malignancies.

## 1. Introduction

In the immune system, each antibody recognizes a specific target antigen through the antibody-antigen binding site, formed by 3 structurally hypervariable loops in the light (*V*_L_) and heavy chain (*V*_H_) variable regions of an Immunoglobulin (Ig), called complementarity determining regions (CDRs).

Antibodies for biomedical purposes can be produced in different formats thanks to the recombinant DNA technology [1]. One of the most used formats is the single-chain variable antibody fragment (scFv), including the Ig* V*_H_ and* V*_L_ domains linked by a flexible polypeptide linker. A scFv retains the specificity towards its target and has increased tissue penetrance, faster blood plasma clearance, and lower immunogenicity with respect to the parental antibody [2, 3]. The ease of manipulation and expression on a large scale make the scFv an ideal antibody format for diagnostic and therapeutic application even in the oncologic field [4].

A number of human cancers are etiologically related to persistent infection with high-risk Human Papillomaviruses (HR HPVs). Worldwide, approximately 600,000 cases of HPV-related diseases occur per year, which still represents a serious problem of public health. Among all, cervical cancer (CC) is predominant and represents the fourth most common cancer in women [5]. HPV16 and HPV18 are the most oncogenic HPV genotypes, causing about 60% and 15% of the CC cases, respectively [6, 7]. Nevertheless, the HPV-associated head and neck cancers increased considerably over the past two decades, especially in developed countries [8, 9].

Three different prophylactic vaccines are now available to prevent infections from 2 to 7 HR HPV genotypes [10, 11]. In spite of their prevention efficacy and long-term protection, a real reduction of CC incidence will not occur before some decades [12]. In fact, HPV vaccines do not affect preexisting infections and do not prevent malignant progression. Also, a total coverage is difficult to achieve especially in developing countries where HPV is endemic. Therefore, development of therapeutic strategies for the treatment of HPV-associated tumor lesions is still a priority.

HPV carcinogenesis depends on the expression of two viral oncoproteins, E6 and E7, acting synergistically to immortalize and transform the infected cells [13, 14].

In view of the p53 critical role in cell cycle and maintenance of host genome integrity, the E6-mediated p53 degradation is a crucial step in cancer development [15]. Accordingly, transgenic mice expressing E6 in the skin develop malignant skin tumors, whereas the E7 expression induces primarily benign hyperplasia [16].

Recently, we selected the scFvI7, specific for the HPV16 E6 oncoprotein (16E6), from the high-diversity murine naive Single Pot Library of Intracellular Antibodies (SPLINT) by Intracellular Antibody Capture Technology (IACT) [17]. The I7 sequence was provided with a tripartite nuclear localization signal (NLS) and expressed as I7nuc intracellular antibody (intrabody) in HPV16-positive cells. Such expression reduced cell proliferation while inducing apoptosis. This effect can be ascribed to p53 rescue. A remarkable hindering effect on tumor onset due to I7nuc expression was observed also in two preclinical mouse models for HPV-associated tumors [18].

We are currently evaluating the antitumor activity of I7nuc in a therapeutic setting for experimental HPV tumors. However, in view of either a safe therapeutic application in humans or a possible use in early diagnosis of HPV infection, characterization of the anti-16E6 scFv in protein format could be extremely valuable.

In this study, we report expression, purification, and characterization of the anti-16E6 scFvI7 and scFvI7nuc. Due to the selection method and previous experiments [18], we know that I7 and I7nuc are specific and can bind to the 16E6 when expressed in eukaryotic environment. We do not know whether the same binding capacity is retained by the scFv produced in prokaryotes as a recombinant protein. Therefore, E6 binding was evaluated by different approaches in parallel with scFv stability, which represents a key feature for both diagnostic and therapeutic applications. With this perspective, the antiproliferative activity of scFvs delivered to HPV-positive cells was also analyzed.

## 2. Materials and Methods

### 2.1. Construction of I7 and I7nuc pQE30 Plasmids and Bacterial Transformation

The anti-16E6 scFv sequences were cloned in the pQE30 prokaryotic vector by different procedures. The I7 sequences were PCR-amplified (95°C for 1 min, 50°C for 1 min, and 74°C for 1 min, 35 cycles) from the eukaryotic vector selected by IACT, using the anti-E6Dir and anti-E6Rev couple of primers to introduce the BamHI and the HindIII restriction sites and cloned in the pGEMT vector, obtaining the I7pGEMT plasmid and then BamHI/HindIII digested. The purified BamHI/HindIII fragment was cloned in pQE30, obtaining I7QE plasmid.

The primer sequences with the restriction sites underlined are as follows:

anti-E6Dir: 5′ GCGCGGATCCGATATTGTGATGACCCAGTC 3′

anti-E6Rev: 5′ GCGCAAGCTTGCGGCCGCAGTACTATCCAGGCCCAG 3′

The I7nuc sequences were PCR amplified as above from the I7nucpGEMT plasmid previously described [18] using the sense primer anti-E6Dir reported above and the I7nucSacRev antisense primer to introduce the BamHI and SacI restriction sites, with sequence as follows:

I7nucSacRev: 5′GCAGGTCGACCATATGGGAGAGCTCCCAA3′ (SacI restriction site underlined)

The purified BamHI/SacI fragment was cloned into pQE30, obtaining I7nucQE plasmid. The PCR products were gel-purified by GFX PCR DNA and Gel Band Purification Kit (GE Healthcare, Buckinghamshire, UK).

For transformation, highly competent* E. coli* NEB Turbo (Biolabs, Ontario, CA) cells were used, and the positive clones were identified by PCR and enzymatic restriction. The chosen clone was then checked by sequence analysis.

Both I7QE and I7nucQE contain a MGRS 6xHis tag at their N-terminus for purification by Ni-NTA affinity chromatography (Ni-NTA resin, QIAGEN, Hilden, Germany).

### 2.2. Expression, Purification, and Refolding of scFv Proteins

*E. coli* cells transformed with pQE30 vectors were grown overnight (ON) in cultures in Luria Bertani (LB) broth in the presence of 2% glucose and ampicillin (100 *μ*g/ml) at 37°C with shaking. ON culture (25 ml) was used to inoculate 500 ml of LB broth and grown until OD_600_ was 0.6. The culture was grown for further 4 h after addition of 2 mM Isopropyl *β*-d-thiogalactopyranoside (IPTG, bioWORLD, USA). Bacterial cells were harvested by centrifugation at 6000 rpm in Sorval GSA rotor for 20 min at room temperature (RT) and lysed in 25 ml of lysis buffer (100 mM NaH_2_PO_4_, 300 mM NaCl, 10 mM Tris-HCl, 5% glucose, and 1 mM DTT, pH 6.3) containing 6 M Guanidine-HCl (EuroClone, Italy). Bacterial suspension was stirred for 30 min at RT followed by intermittent ultrasonication on ice for 20 min (Vibra Cell, Sonics & Materials Inc., Newton, USA). Afterwards, the lysate was centrifuged at 10000 rpm in Sorval SS34 rotor for 30 min at 4°C, and supernatant was used for affinity‏ chromatography purification on Ni-NTA resin according to QIAexpressionist (QIAGEN). In brief, 1 ml of prewashed resin was added to supernatant into 50 ml Falcon tube and incubated at RT for 30 min while mixing. The protein-resin mixture was recovered by centrifuging at 1500 rpm for 5 min and washed repetitively in 250 ml of denaturing buffer (100 mM NaH_2_PO_4_, 10 mM Tris-HCl, and 8 M Urea) at pH 6.3 until OD_280_ of the waste was 0.002. The resin was then packaged into a 1 ml polypropylene column and the captured scFv eluted with denaturing buffer at pH 5.9 (4 fractions) and pH 4.5 (4 fractions, 1 column volume each). To evaluate scFv purity, an aliquot of each fraction added with SDS-loading buffer (25 mM Tris-HCl pH 6.8, 5% *β*-Mercaptoethanol, 2% SDS, 50% glycerol) was analyzed by 12% SDS-PAGE and Coomassie Brilliant Blue (CBB) staining.

The scFv-containing fractions were pooled and subjected to stepwise dialysis into a Slide-A-Lyzer cassettes (10 MWCO, Thermo Fisher Scientific, Rockford, IL) for 24 h at 4°C against 500 ml of renaturing buffer (rB: 50 mM NaCl, 25 mM Tris-HCl, 0.25 M glucose, and 10% glycerol, pH 7.0) containing decreasing concentrations of urea (from 6 M to 0) at 4°C. The I7 and I7nuc proteins concentration was determined using Bradford assay (Bio-Rad, Ontario, CA) with Bovine Serum Albumin (BSA, Biolabs) as a protein standard.

### 2.3. ELISA Microtiter Binding Assay

A microtiter 96-well plate (Nunc MaxiSorp, Thermo Fisher Scientific) was coated with 350 ng/well of recombinant 16E6 [19] or BSA in 50 mM carbonate/bicarbonate buffer, pH 9.4 (Thermo Fisher Scientific). The plate was saturated with 200 *μ*l/well of 2% Non-Fat Dry Milk (NFDM, Bio-Rad) PBS buffer at 37°C for 2 h and incubated 1 h at 37°C with 100 *μ*l/well of scFvI7 or scFvI7nuc in PBS (2.5 *μ*g/ml). An in-house anti-16E6 mouse polyclonal IgG 1 : 1000 in 2% NFDM-PBS was used as a positive control [19]. The wells were rinsed with PBS for 3 times and incubated with 50 *μ*l/well of mouse anti-V5 tag monoclonal antibody (mAb, Life Technologies, USA) 1 : 2500 in 2% NFDM-PBS 1 h at 37°C. After repeated washes, wells were incubated with 100 *μ*l of anti-mouse horseradish peroxidase- (HRP-) conjugated polyclonal IgG (Sigma-Aldrich, USA) 1 : 20000 in 2% NFDM-PBS, 1 h at 37°C. After a final wash, color was developed using the TMB substrate kit (Vector Laboratories, Burlingame, CA). The reaction was stopped after 2 h with 100 *μ*l of 2 M H_2_SO_4_ and OD_450_ determined in a microtiter plate reader (iMark, Bio-Rad).

### 2.4. Western Blotting

ScFvI7 and scFvI7nuc (2 *μ*g/ml) were incubated in 0.2% human serum albumin (HSA, Sigma-Aldrich) PBS at 37°C. At different time intervals, aliquots of 500 ng were analyzed by 12% SDS-PAGE. The proteins were transferred onto 0.4 *μ*m pore-size polyvinylidene difluoride membrane (PVDF, Millipore, Billerica, MA, USA). Unspecific binding sites were blocked for 2 h at RT in 5% NFDM-TBS buffer. ScFvs were detected by mouse anti-V5 tag mAb 1 : 500 in TBS ON at 4°C, followed by anti-mouse HRP-conjugated polyclonal IgG 1 : 10000 in 3% NFDM-TBS and enhanced chemiluminescence substrate solution (ECL, Millipore, Billerica, MA, USA) using BioSpectrum Imaging system (UVP, CA) and the software program UVP (VisionWorks LS, Analytik Jena Company).

### 2.5. Slot Blotting

Twofold serial dilutions in TBS of recombinant 16E6 [19] or BSA in the range of 62.5–500 ng were spotted onto 0.2 *μ*m Protran nitrocellulose membrane (Whatman, Dassel, Germany) assembled in the Bio-Dot SF microfiltration apparatus (GE Healthcare Life Sciences, Piscataway, NJ) according to the manufacturer's instructions. After extensive washing with TBS, the membrane was blocked in 5% NFDM-TBS and divided into strips and then incubated ON at 4°C with scFvI7 or scFvI7nuc in TBS (10 or 20 *μ*g/ml). The in-house anti-16E6 mouse polyclonal IgG 1 : 500 in 3% NFDM-TBS was used as a positive control [19]. After washing 3 times in TBS, the membrane was incubated with mouse anti-V5 tag mAb 1 : 500 in TBS followed by anti-mouse HRP-conjugated polyclonal IgG 1 : 10000 in 3% NFDM-TBS. After extensive washing with TBS, the 16E6-scFv complexes were detected by incubation with ECL-substrate solution and BioSpectrum Imaging system using the software program UVP.

### 2.6. Cells, Immunofluorescence, and Confocal Analysis

The human cervical carcinoma SiHa keratinocytes (ATCC HTB-35) harboring the HPV16 genome and expressing the E6 and E7 proteins were grown at 37°C in humidified atmosphere with 5% CO_2_ in DMEM (Gibco, UK) with 10% heat-inactivated Fetal Bovine Serum (Corning, USA), 1 unit/ml penicillin, 1 *μ*g/ml streptomycin, and 2 mM glutamine.

SiHa cells (3 × 10^5^) were seeded in 35 mm dishes and grown for 24 h at 37°C and then fixed with 3.6% paraformaldehyde (Sigma-Aldrich) PBS for 20 min at 4°C and permeabilized with 0.25% Triton X-100-PBS for 10 min. After blocking with 1% NFDM-PBS for 10 min at RT, the cells were incubated for 1 h at RT with scFvI7 or scFvI7nuc (2 *μ*g/ml) or an in-house anti-16E6 rabbit polyclonal IgG 1 : 300 [19]. The antigen-antibody complexes were detected by mouse anti-V5 tag mAb 1 : 500 followed by a fluorescein-conjugated goat anti-mouse IgG (GAM-FITC, ICN Cappel Inc., Ohio, USA) 1 : 400 or Alexa-Fluor 594 goat anti-rabbit IgG (Life Technologies, USA) 1 : 200.

For double detection of E6, consecutive incubations with rabbit anti-16E6 polyclonal IgG followed by Alexa-Fluor 594 goat anti-rabbit IgG and with purified scFv followed by mouse anti-V5 mAb and GAM-FITC were performed in the same well. Preimmune rabbit serum was used 1 : 300 as a negative control. All the antibodies were diluted in 1% NFDM-PBS. RedDot™2 dye (Biotium, Inc., Hayward, USA) 1 : 400 in PBS was utilized as a nuclear marker. All samples were examined using a confocal microscope Leica TCS SP5 and processed with LAS AF version 1.6.3 software (Leica Microsystems). To prevent cross emission spectra, specific lasers (488 nm, 546 nm, and 633 nm) were activated in sequential mode to acquire the images.

### 2.7. Delivery of scFvs and Analysis of Cell Proliferation

For scFvs delivery, SiHa cells (5 × 10^5^) seeded in 35 mm dishes and grown in complete medium up to 50–60% of confluence were washed and treated with 500 *μ*l of DMEM containing the purified scFv at a final concentration of 2.5 *μ*g/ml. Cells incubated with 500 *μ*l of DMEM containing the same volume of rB served as controls. After 6 hours, scFv entry was detected by immunofluorescence using mouse anti-V5 tag mAb as described above and visualized using FLoid Cell Imaging Station microscope (Thermo Fisher Scientific, USA).

To analyze the effect of antibody delivery, SiHa cells were seeded (10,000 cells/well) in a 96-well plate (Corning Inc., USA). After 24 hours, cells were washed and medium was replaced with 50 *μ*l of DMEM containing the scFvs (2.5 *μ*g/ml in rB) or rB alone as a control. To exclude any possible interference by the impurities contained in the antibody preparation, SiHa cells were also incubated with 50 *μ*l of the same DMEM/rB solution, previously scFv-depleted by protein A incubation. After 6 hours at 37°C, the different solutions were replaced with complete DMEM. Cell viability was determined 48 hours after delivery by MTS assay (CellTiter 96® AQ_ueous_ One Solution Cell Proliferation Assay, Promega, Madison, USA) and densitometry analysis according to manufacturer's instructions. Alternatively, 48 h after scFv delivery, cells survival was analyzed by cell forming assay (CFA) as previously described [18].

### 2.8. Statistical Analysis

Data were expressed as the mean ± standard deviation (SD). Statistical analysis was performed using Student's *t*-test for unpaired data and *p* values > 0.05 were considered not significant.

## 3. Results

### 3.1. Design, Cloning, and Prokaryotic Expression of Anti-16E6 scFvs 

The I7 sequence was amplified by specific primers from the plasmid originally selected by IACT, to be cloned, through pGEMT plasmid, in the BamHI/HindIII restriction sites of the pQE30 vector, obtaining the I7QE plasmid.

The I7nuc sequence was amplified by specific primers from the I7nucpGEMT previously described [18] and cloned in pQE30 BamHI/SacI restriction sites, obtaining I7nucQE. The plasmids obtained were checked by sequencing.

**Figure 1:**
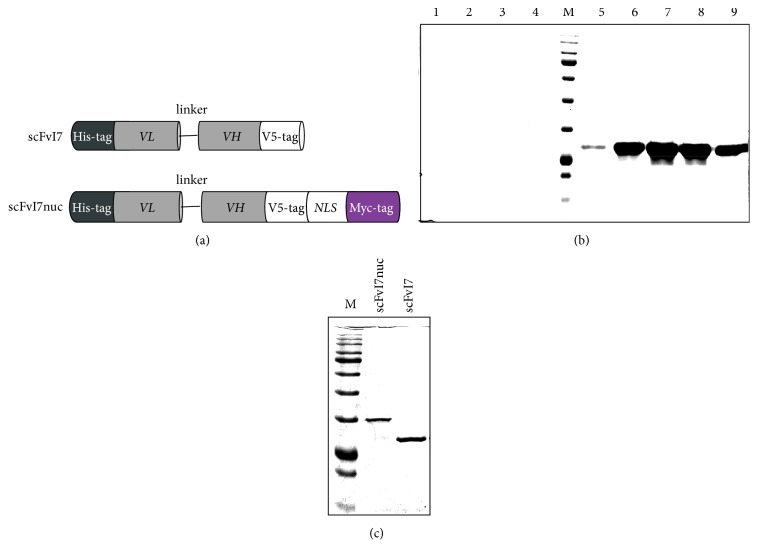
Expression and purification of anti-16E6 scFvs. (a) Schematic representation of the scFvI7 and scFvI7nuc proteins. Light chain (*V*_L_), polylinker, and heavy chain (*V*_H_) domains are shown. N-terminal His-tag and C-terminal V5 tag allow purification and immunodetection, respectively. The SV40 nuclear localization signal and Myc-tag present in scFvI7nuc are indicated. (b) Purification of anti-16E6 scFvs by Ni-NTA chromatography. SDS-PAGE analysis and CBB staining of aliquots of the scFvI7nuc eluates obtained at pH 5.9 (lanes 1–4) and pH 4.5 (lanes 5–9). (c) SDS-PAGE analysis and CBB staining of scFvI7nuc and scFvI7 after purification and refolding. M: protein markers.

The scFvI7 and scFvI7nuc proteins expressed from the plasmids described are schematically represented in Figure 1(a).

The expression of scFvI7 and scFvI7nuc in* E. coli* was optimized on a small scale through a time-course study under 2 mM IPTG induction. The scFv product present in bacteria pellet was evaluated by SDS-PAGE before and after IPTG induction. Although a faint band corresponding to scFvI7 (29 kDa) and scFvI7nuc MW (34 kDa) was already present before IPTG induction, the proteins accumulated rapidly and reached the maximum expression at 4 h, which was the time chosen for scFv production on a larger scale (data not shown).

Different purification procedures were preliminarily performed to obtain the scFvs in a soluble form and with a high degree of purity. Neither protein could be purified using a native protocol because of contaminants present in the soluble fractions. Conversely, a high grade of purity was achieved using a denaturing protocol as described in Materials and Methods. Elution was tentatively performed at pH 5.9 and then continued at pH 4.5. For both scFvs, elution was virtually absent at pH 5.9 and definitely abundant at pH 4.5, which allows for high degree of 6xHis-tagged protein purification (QIAexpressionist).  Figure 1(b) shows the results obtained during scFvI7nuc purification. The purified scFvs were subjected to refolding by stepwise dialysis in decreasing concentration of urea and analyzed by 12% SDS-PAGE and CCB staining (Figure 1(c)). For both scFvs, the total yield ranged from 1 to 4 mg/L of bacterial culture.

### 3.2. Determination of Specificity and Sensitivity of scFvI7 and scFvI7nuc towards the 16E6

**Figure 2:**
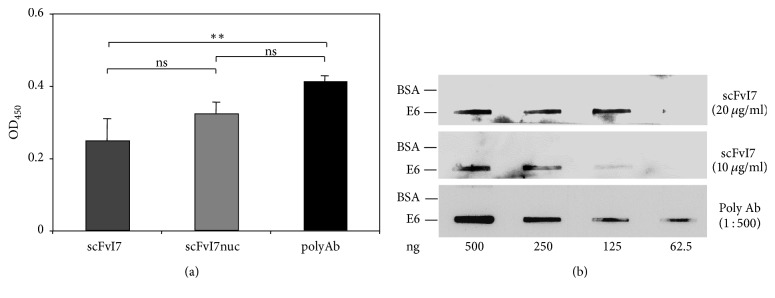
Characterization of the scFvs binding to 16E6. (a) Specificity of I7 and I7nuc scFvs binding to recombinant 16E6 in ELISA. Three experiments were performed and similar results were obtained. Data represent the mean ± SD of samples in quadruplicate from a representative experiment. ^*∗∗*^*p*< 0,01; *p* > 0,05 is considered not significant (ns). (b) Sensitivity of scFv binding to recombinant 16E6 in slot blotting analysis. Serial dilutions of the recombinant 16E6 protein or BSA as a negative control were incubated with the indicated concentrations of scFvI7. Anti-16E6 polyclonal antibody was used as a positive control in (a) and (b).

The specificity of scFvI7 and scFvI7nuc was analyzed in ELISA using the recombinant 16E6. To perform the assay, scFvI7 and scFvI7nuc at 2.5 *μ*g/ml were added to the wells coated with 16E6 or BSA. Both scFvs bound to their antigen specifically even though the scFvI7 signal revealed in ELISA was weaker. Interestingly, we observed similar binding profiles for scFvI7nuc and an anti-16E6 mouse polyclonal IgG (Figure 2(a)).

To analyze the anti-16E6 scFvs sensitivity by slot blot analysis, the recombinant 16E6 and BSA were applied in serial dilutions to nitrocellulose membrane and probed with two identical concentrations of purified scFvI7 or scFvI7nuc. ScFvI7 at a concentration of 10 *μ*g/ml was able to detect up to 125 ng of E6 per slot, showing sensitivity similar to that of the polyclonal antibody, while no signal was observed in the BSA slots (Figure 2(b)). Similar results were obtained with scFvI7nuc (data not shown).

### 3.3. Determination of the scFv Stability In Vitro

**Figure 3:**
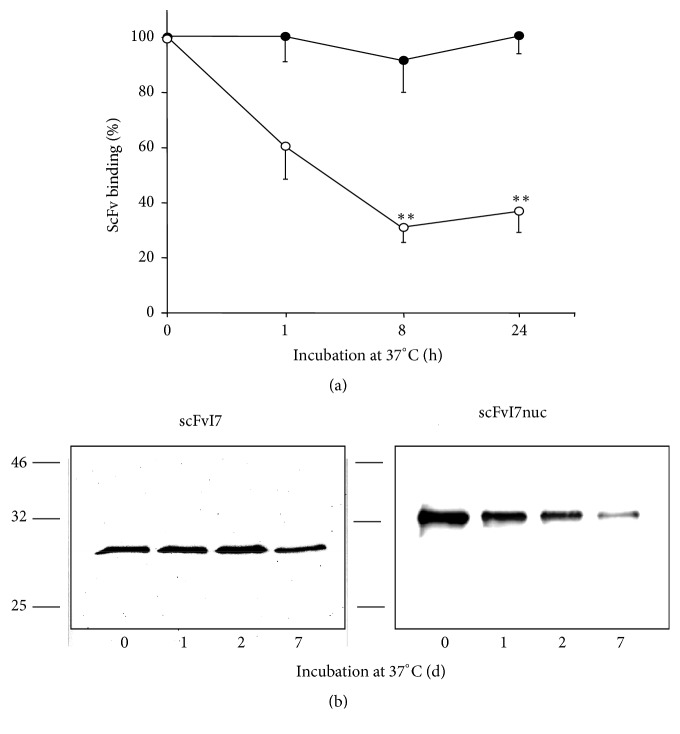
Stability of the anti-16E6 scFv proteins. (a) Residual E6-binding activity of the purified scFvI7 (filled circle) and scFvI7nuc (empty circle) was analyzed in ELISA after incubation at 37°C for different time intervals. Data are expressed as a percentage of the antibody binding without incubation at 37°C and represent the mean ± standard deviation (SD) of three independent experiments. ^*∗∗*^*p* < 0.01. (b) Kinetics of scFv protein degradation at 37°C were analyzed in WB.

An adequate resistance to physiologic temperature is essential for the scFvs successful employment. Therefore, we investigated the scFvs thermal resistance by analyzing in ELISA the binding to their antigen after different incubation times at 37°C in the presence of 0.2% HSA to mimic a physiologic environment. As shown in Figure 3(a), while scFvI7 binding activity to E6 remained constant for the time of analysis (24 h), scFvI7nuc binding activity decreased to 33% after only 8 h of incubation. The different degradation profile of the two proteins was confirmed in Western blotting, where incubation of scFvs with HSA at 37°C caused a gradual but sharp decrease of the scFvI7nuc amount with respect to scFvI7, which was clearly reduced after 1 week (Figure 3(b)). The reason for such difference in stability between the two scFvs is not clear as they differ from each other only in the presence of the myc-tag and tripartite NLS in scFvI7nuc. However, the presence of these peptide sequences enhances the pI from 9.45 to 9.83, suggesting that the increase of repulsive forces due to positively charged amino acids could alter conformational stability of the protein [20]. Our findings are in agreement with a paper that analyzed the NLS involvement in protein stability in vitro, showing that a highly basic NLS can severely compromise the scFv expression from the host parental vector [21].

### 3.4. Analysis by Immunofluorescence and Confocal Microscopy of scFvI7 and scFvI7nuc Binding to E6 in HPV16-Positive SiHa Cells

The ability of the purified scFvs to recognize the endogenous 16E6 was analyzed in the human HPV16-positive SiHa cells by immunofluorescence and confocal microscopy analysis. 

**Figure 4:**
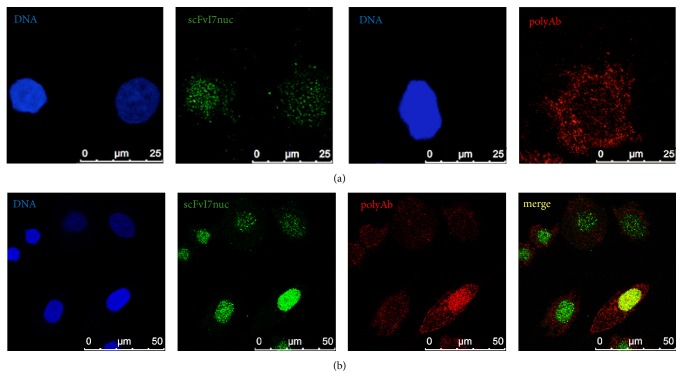
Detection of endogenous 16E6 protein by scFvI7nuc in confocal microscopy. SiHa cells were stained with purified scFvI7nuc (green) and anti-16E6 polyclonal IgG (polyAb, red) used alone (a) or in combination (b). Cell nuclei were counterstained using RedDot™2 dye and are displayed in blue. Yellow staining in the "merge" image indicates detection of nuclear E6 by both antibodies. Samples were analyzed using confocal microscope (Leica TCS SP5). Software: LAS AF version 1.6.3 (Leica Microsystems). Image magnification: 3938x in panel (a) and 1969x in panel (b).

The purified scFvI7nuc was able to detect endogenous E6 expressed in the nucleus of SiHa cells (Figure 4(a)). As a positive control, we used an anti-16E6 rabbit polyclonal IgG [19]. Surprisingly, confocal microscopy analysis showed that while the polyclonal Ab (red) could detect the E6 in both the nucleus and cytoplasm of SiHa cells, the purified scFvI7nuc (green) was able to detect mainly a nuclear form of E6. ScFvI7 worked similarly to scFvI7nuc (data not shown).

To exclude the fact that subcellular distribution could depend on a different E6 expression in unsynchronized cell cultures, we analyzed the E6 localization using the polyclonal Ab and each scFv in turn in the same well. We could observe overlapping of red and green staining (yellow) only in the cells showing particularly high intranuclear E6 levels, while in most cells, scFvI7nuc decorates E6 in the nucleus (Figure 4(b)). In this regard, we can speculate that our scFvs recognize an E6 epitope that is exposed only by the intranuclear E6, while the polyclonal Ab, reasonably recognizing several epitopes, would be able to detect all the E6 forms.

Our findings are consistent with the observation that the HR HPVs E6 exist in monomeric and oligomeric forms, which expose different conformational epitopes. Interestingly, other authors showed a diffuse distribution of E6 homo- or heterooligomers in HPV-transformed cells, while the monomeric oncoprotein was present mainly in the cell nucleus [22].

It is known that splicing events regulate the translation of full-length and truncated forms of E6 in HR HPV-infected cells. Such different isoforms retain different functions [23] and show different intracellular distribution, and the full-length form is expressed preferentially in cell nucleus [24, 25]. However, regardless of the nature of the nuclear E6 recognized by the scFvs, it could represent the biologically active protein, since the I7nuc intrabody expression, by perturbing the E6 interactions with cellular targets, resulted in decreased proliferation and survival of HPV16-positive cells [18].

### 3.5. Cellular Uptake of scFvI7nuc Protein

We have recently shown that intracellular I7nuc expression hampers the development of HPV16-positive tumors in animal models [18].

In view of potential therapeutic applications, the study of the impact of anti-E6 antibodies delivery to SiHa cells is of particular interest.

**Figure 5:**
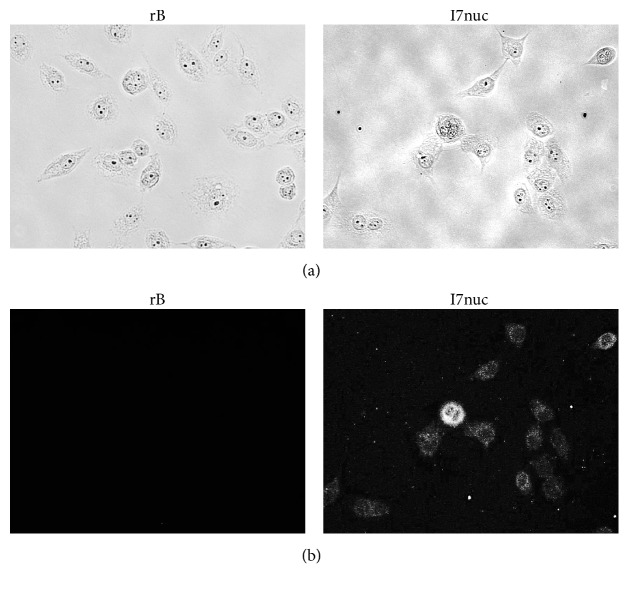
Internalization of scFvI7nuc protein in SiHa cells. SiHa cells were incubated with DMEM containing rB with (I7nuc) or without (rB) the scFvI7nuc. After 6 hours of treatment, the internalized antibody was visualized using mouse anti-V5 tag mAb followed by secondary antibody conjugated with fluorescein. Microscopy images (a) and immunofluorescence images (b) showing the scFvI7nuc internalization into SiHa cells. Images were captured using FLoid Cell Imaging Station microscope (Thermo Fisher Scientific, USA). Magnification: 600x.

It is well known that antibodies are poorly transported across cell membranes, and different delivery strategies are investigated with particular regard to safety, necessary for clinical use. Such strategies include vehiculation by liposomes, nanoparticles, and extracellular vesicles [26] as well as the enhancement of the isoelectric point (PI) of a protein molecule by fusion to cationic peptides known as cell-penetrating peptides or chemical derivatization of surface carboxyl groups, generating primary amino groups [27]. In particular, protein cationization has been proven to be a simple and effective method to deliver functional antibodies into cells [28]. Since our scFvs have themselves an intrinsically high PI, we explored the possibility to deliver them directly into SiHa cells. After 6 hours of incubation with the antibody solution or control solution, the scFvs entry was checked by immunofluorescence. As shown in Figure 5(b), scFvI7nuc uptake was efficient, as virtually all the cells resulted to be fluorescent. However, different from intranuclear distribution of I7nuc expressed as an intrabody [18], the localization of endocytosed scFvI7nuc was mainly cytoplasmic and, only faintly, nuclear. Although we do not actually know whether the limited nuclear localization observed is ascribable to experimental conditions or to the documented instability of scFvI7nuc at 37°C, this result demonstrates that the endocytosed antibody retains the ability of nuclear entry.

Different from scFvI7nuc, scFvI7 entered scarcely (data not shown). We believe this could be ascribed to the different PIs of the two proteins, suggesting that even a slight difference related to this parameter might affect the interaction with the cell membrane, thus precluding the adsorptive-mediated endocytosis of the molecule.

To study the scFvI7nuc effect on cell viability, SiHa cells were observed at different times after delivery. Interestingly, after ON incubation, scFvI7nuc-receiving cells were suffering and were in a lower number with respect to the control cells (data not shown). To confirm this observation and investigate the potential antiproliferative effect of scFvI7nuc, we analyzed the impact of the antibody administration on proliferation and survival of HPV16-positive cells by MTS. At 48 h after uptake, we observed a sharp decrease, in the range of 30–60%, of formazan conversion in cells treated with scFvI7nuc with respect to the control cells. Instead, no reduction of cell viability was observed after treatment with either rB or the scFv-depleted rB solution in DMEM. This finding confirms that I7nuc in protein format maintains a biological activity similar to that associated with the I7nuc intrabody expression [18]. The antiproliferative effect was also confirmed in CFA performed 48 h after scFvI7nuc administration, showing a reduction of colony number in treated cells equal to 72% (67 colonies ± 14/1000 plated cells) with respect to untreated SiHa cells (240 colonies ± 10/1000 plated cells).

## 4. Conclusions

The aim of this study was to produce and characterize E6-specific scFvs as potential tools for the therapy of HPV16-associated lesions.

We reported successful cloning, expression, and purification of single-chain antibody fragments directed towards 16E6. We demonstrated the ability of the purified antibody fragments to detect 16E6 in different assays. First, we used ELISA and slot blotting to show the interaction of scFvI7 and scFvI7nuc with the recombinant 16E6. Next, we confirmed by immunofluorescence the ability of both scFvs to bind to E6 in immortalized HPV16-positive cells. Furthermore, we checked for stability of the scFv molecules as an essential feature for future applications. Lastly, we verified the ability of scFvI7nuc protein to hamper viability and proliferation of HPV16-positive cells* in vitro*.

Our results demonstrate that the purified scFvs retain specificity, sensitivity, and sufficient stability for detection of E6 oncoprotein* in vivo* and could pave the way for the development of novel, safe therapeutic tools specifically targeting the HPV16 oncoprotein.

## Data Availability

The data used to support the findings of this study are available from the corresponding author upon request.
